# Para‐Aortic Lymph Node Metastasis Detected by Sentinel Lymph Node Biopsy in Cervical Cancer Initially Presumed to be Stage IA2: A Case Report

**DOI:** 10.1111/jog.70377

**Published:** 2026-06-19

**Authors:** Hatsune Ouchi, Tatsuyuki Chiyoda, Kensuke Sakai, Yutaka Shiraishi, Hiroshi Nishio, Wataru Yamagami

**Affiliations:** ^1^ Department of Obstetrics and Gynecology Keio University School of Medicine Tokyo Japan; ^2^ Department of Radiology Keio University School of Medicine Tokyo Japan

**Keywords:** cervical cancer, micrometastasis, para‐aortic lymph node, sentinel node, ultrastaging

## Abstract

Sentinel node navigation surgery (SNNS) minimizes unnecessary lymphadenectomy and its associated complications while enhancing detection of lymph node micrometastases through ultrastaging. A 34‐year‐old nulligravid woman with a history of CIN2 was diagnosed with squamous cell carcinoma (4.5 mm width, 4 mm depth) with lymphovascular invasion after conization. Clinical stage was T1a2 (FIGO 2018). Modified‐radical trachelectomy with SNNS was planned for fertility preservation. SPECT–CT identified bilateral obturator and left lower para‐aortic sentinel nodes. Intraoperative frozen sections revealed a 1.1 mm micrometastasis in the left obturator node and a 2.5 mm macrometastasis in the left para‐aortic node. The procedure was converted to modified‐radical hysterectomy with bilateral adnexectomy and pelvic lymphadenectomy, followed by concurrent chemoradiotherapy including the para‐aortic field. The patient remains disease‐free at 10 months. Without SNNS, the para‐aortic metastasis would have been undetected, resulting in inadequate radiation coverage. SNNS fundamentally changes therapeutic approaches by enabling accurate disease extent identification and individualized treatment.

## 1 Introduction

Among cervical cancer patients with FIGO 2009 Stages IA–IIA disease, lymph node metastases occur in only 14.8% [1]. Sentinel node navigation surgery (SNNS) has been adopted in gynecologic oncology to reduce unnecessary lymphadenectomy and associated complications such as lymphedema, lymphocele, and lymphatic abscess [2]. Additionally, ultrastaging pathological evaluation enables more accurate detection of lymph node metastases [2]. We present a case of presumed Stage IA2 (FIGO2018) cervical cancer in which SNNS identified a para‐aortic lymph node metastasis. Although the case was clinically presumed preoperatively to be Stage IA2 (FIGO 2018), final pathological staging revealed pT1b1 N2 M0, corresponding to Stage IIIC2 (FIGO 2018). This illustrates how SNNS may identify both occult tumor extension and unsuspected nodal disease in cases initially considered to be very early stage. Importantly, the growing interest in fertility‐sparing and de‐escalated surgical approaches for presumed FIGO Stage IA cervical cancer heightens the need for highly accurate preoperative staging and careful assessment of occult disease extent before such conservative management can be safely offered.

## 2 Case Presentation

A 34‐year‐old nulligravid woman with no significant medical or family history had been followed for CIN2 until 4 years prior, when she discontinued follow‐up. During fertility workup, cervical cytology showed HSIL, and biopsy revealed CIN3. She underwent conization, which demonstrated HPV‐associated squamous cell carcinoma with lymphovascular invasion, measuring 4.5 mm in width and 4 mm in depth. Surgical margins were positive for CIN1‐2. She was referred for fertility‐sparing surgery. The conization had been performed at the referring hospital as therapeutic management of biopsy‐proven CIN3, rather than as a staging procedure for an established invasive carcinoma; consequently, no preconization MRI had been obtained.

Postoperative cytology was negative (NILM) and biopsy showed only atypical epithelia. MRI revealed no residual disease (Figure 1), and PET‐CT showed no metastases. Based on T1a2 (FIGO2018) disease with lymphovascular invasion, modified‐radical trachelectomy (Piver Type II) with SNNS was planned.

**FIGURE 1:**
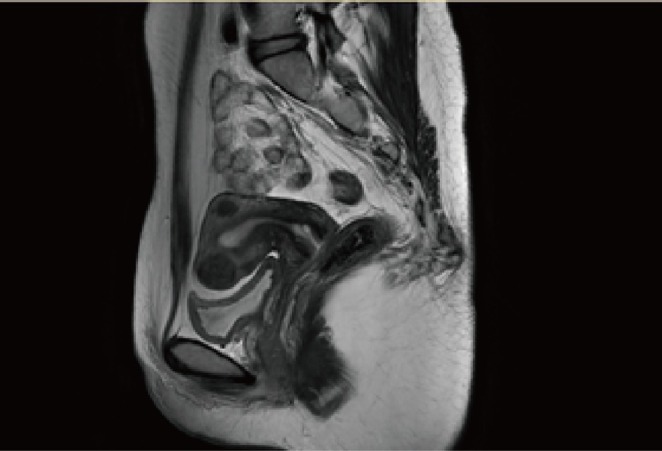
Preoperative MRI. No obvious residual lesion is identified. Multiple intramural myomas are present without malignant findings. Sagittal T2‐weighted image.

On the day before surgery, 57.2 MBq 99mTc‐phytate was injected into the cervix at 2, 4, 8, and 10 o'clock positions. SPECT–CT on the morning of surgery identified bilateral obturator and left lower para‐aortic lymph nodes as sentinel nodes (Figure 2).

**FIGURE 2:**
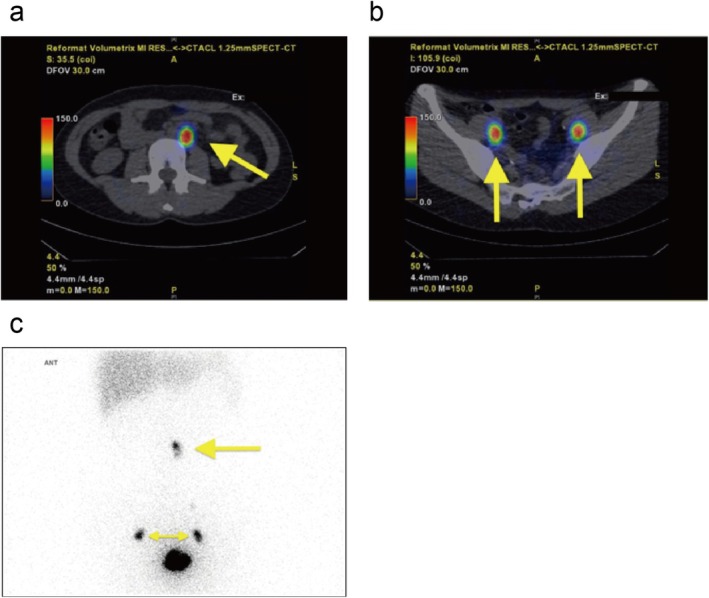
SPECT–CT. (a) Left lower para‐aortic lymph node showing RI accumulation (arrow). (b) Bilateral obturator lymph nodes showing RI accumulation (arrow). (c) Coronal view showing accumulation in left lower para‐aortic node (arrow) and bilateral obturator nodes (double arrows).

Intraoperatively, no abnormal findings were observed except for intramural myomas. Gamma probe counts were right obturator node 22 410 counts per second (cps), left obturator node 17 860 cps, and left lower para‐aortic node 6881 cps. Sentinel nodes showed no gross enlargement. Frozen section analysis with short‐axis 2 mm interval sectioning revealed a 2.5 mm macrometastasis in the left lower para‐aortic node and a 1.1 mm micrometastasis in the left obturator node (Figure 3a,b). The right obturator node was negative. Following the intraoperative confirmation of lymph node metastases, fertility‐sparing surgery was no longer oncologically appropriate, and the procedure was converted to modified‐radical hysterectomy with bilateral salpingo‐oophorectomy and pelvic lymphadenectomy, in accordance with the surgical plan predetermined in consultation with the patient.

**FIGURE 3:**
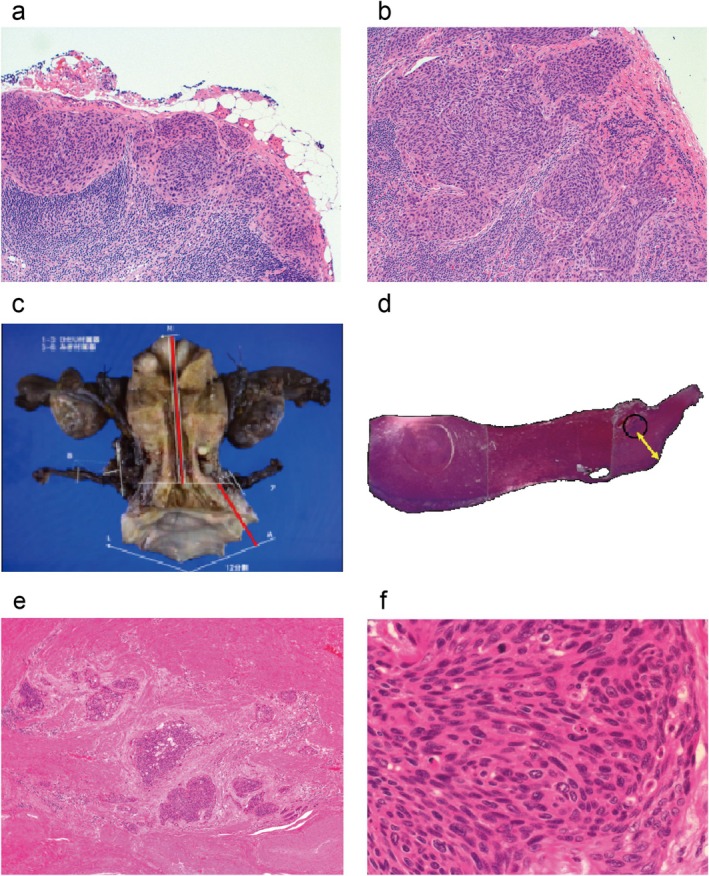
Pathological findings. (a) The left lower para‐aortic node contained a 2.5 mm metastasis. H&E staining, ×100 magnification. (b) H&E staining of a 1.1 mm micrometastasis in the left obturator node, ×100 magnification. (c) Sampling diagram. (d) Macroscopic image showing lesion 7–9 mm from cervical canal (double arrows), 2 mm in width (circle). (e) H&E staining of cervical lesion, ×40 magnification. (f) H&E staining of cervical lesion, ×400 magnification.

Final pathology was assessed by integrating the findings from both the conization and hysterectomy specimens. The conization specimen contained an invasive component measuring 4.5 mm in width and 4 mm in depth, with negative surgical margins. The hysterectomy specimen showed a discontinuous residual tumor located 7–9 mm from the cervical canal, measuring 2 mm in width, at a site anatomically separate from the prior conization margin. Immunohistochemistry was positive for p16, consistent with HPV‐associated keratinizing squamous cell carcinoma (Figure 3c–f). D2‐40 staining confirmed lymphovascular invasion. The final diagnosis was pT1b1 N2 M0, Stage IIIC2 (FIGO 2018).

She was discharged on postoperative day 10. Concurrent chemoradiotherapy (6 cycles of cisplatin with 50.4 Gy/28 fractions volumetric modulated arc therapy to the pelvis and para‐aortic region, plus 56 Gy simultaneous integrated boost to sentinel node‐positive sites) was initiated 41 days postoperatively. She remains disease‐free 10 months posttreatment.

## 3 Discussion

Lymph node metastasis rates in early cervical cancer are influenced by lymphovascular invasion. Without lymphovascular invasion, pelvic node positivity rates are less than 1% for Stage IA1 and 1.7% for IA2. With lymphovascular invasion, rates increase to 7.8% for IA1 and 14.6% for IA2 [3]. Despite the higher risk, this patient showed no metastases on preoperative imaging, highlighting the limitations of conventional imaging modalities.

Beyond the limitations of imaging, the pathological findings in this case also raise the question of whether the initial conization fully captured the extent of the primary tumor. The hysterectomy specimen demonstrated a discontinuous residual lesion located several millimeters apart from the prior conization margin, a distribution that is unusual for truly microinvasive squamous cell carcinoma. We therefore consider it more plausible that the conization underestimated the true tumor extent rather than that this represents a genuine multifocal microinvasive presentation. This interpretation is consistent with the absence of preconization MRI in the patient, since the conization was performed at the referring institution as therapeutic management of biopsy‐proven CIN3 rather than as a staging procedure for an established invasive carcinoma. Taken together, the discontinuous residual disease and the unsuspected nodal metastases reinforce the message that presumed FIGO IA2 disease, particularly when accompanied by lymphovascular invasion, may occasionally harbor biologically more advanced disease than preoperative assessment suggests.

Choi et al.'s meta‐analysis reported lymph node metastasis detection sensitivities of 50% for CT, 56% for MRI, and 82% for PET‐CT, with specificities of 92%, 91%, and 95%, respectively [4]. While PET‐CT demonstrates superior performance, its false‐negative rate remains clinically significant. Gouy et al. found that 8.5%–10% of locally advanced cervical cancer patients with negative preoperative CT, MRI, and PET‐CT imaging had para‐aortic metastases on laparoscopic lymphadenectomy [5]. Although their study focused on locally advanced disease rather than early‐stage cases like ours, it demonstrates the inherent limitations of imaging in detecting lymph node metastases, particularly micrometastases and small macrometastases.

In our case, both the 1.1 mm obturator micrometastasis and 2.5 mm para‐aortic macrometastasis were undetectable by imaging. Standard imaging techniques rely on size and morphological criteria, which may not identify metastases in normal‐sized nodes. This limitation is particularly relevant in early cervical cancer, where small‐volume metastases are more common than bulky nodal disease.

Without SNNS, conventional pelvic lymphadenectomy would not have included para‐aortic node sampling, and the metastasis would have been missed. Subsequent radiotherapy would have been limited to the pelvis, excluding the para‐aortic region. This would have resulted in inadequate treatment of occult metastatic disease, potentially compromising oncologic outcomes. SNNS fundamentally changed the therapeutic approach by accurately identifying the extent of disease.

The ultrastaging protocol, involving short axis 2 mm interval sectioning of sentinel nodes, enabled detection of the 1.1 mm obturator micrometastasis. Standard pathological evaluation with wider sectioning intervals might have missed this finding. Recent studies have demonstrated the value of ultrastaging in detecting occult metastases. The SENTIX prospective international trial reported that ultrastaging detected an additional 43% of node‐positive cases (macrometastases and micrometastases) in patients with negative lymph nodes by imaging and intraoperative assessment [6]. Similarly, a large multicenter study found that ultrastaging identified low‐volume lymph node metastases in 11% of patients that would have been missed by standard pathological processing [7]. This enhanced detection allows for more accurate staging and treatment planning.

Para‐aortic sentinel nodes are identified in 0.4%–3.8% of Stages IA–IIB cervical cancer cases [2, 8]. While uncommon, their identification has significant therapeutic implications. Reports also describe sentinel nodes in unconventional locations such as sacral, common iliac, and inguinal regions. This anatomical variability suggests that lymphatic drainage patterns in cervical cancer are more complex than traditional surgical dogma assumes. In our case, the lymphatic drainage pattern cannot be unambiguously interpreted. Two scenarios are compatible with the findings: (a) direct drainage from the cervix to the para‐aortic nodes, bypassing the pelvic nodes or (b) sequential metastatic spread via the upstream left obturator node, which itself harbored a 1.1 mm micrometastasis. The gamma‐probe activity ratio (obturator stations 17 860–22 410 cps versus para‐aortic 6881 cps) is consistent with sequential drainage but does not exclude direct drainage, given the anatomic distance from the injection site. Conversely, the inverse size relationship—a 2.5 mm para‐aortic macrometastasis with only a 1.1 mm upstream micrometastasis—is unusual for purely sequential progression and is more consistent with at least partially independent drainage pathways. However, biological variability in metastatic kinetics precludes a definitive conclusion. Importantly, this anatomical variability suggests that routine lymphadenectomy of identical regions for all patients may miss metastases at unconventional drainage sites.

This patient initially desired fertility preservation, making SNNS, particularly, valuable for risk stratification. The detection of para‐aortic metastasis precluded fertility‐sparing surgery and indicated the need for definitive chemoradiotherapy. While this outcome was disappointing from a fertility perspective, early detection of advanced disease prevented inadequate treatment that would have resulted from performing trachelectomy without comprehensive nodal assessment. More broadly, this case supports the view that comprehensive nodal assessment with intraoperative pathological backup is essential when fertility‐sparing or de‐escalated surgery is considered for presumed FIGO IA2 disease with lymphovascular invasion. Conservative surgery in this setting should be undertaken only with a clearly predefined backup plan for conversion to definitive treatment if occult nodal disease is identified intraoperatively, as was the case here.

The optimal management of sentinel node‐positive early cervical cancer remains debated. Some advocate for completion lymphadenectomy followed by adjuvant therapy, while others suggest proceeding directly to chemoradiotherapy to avoid surgical morbidity. In our case, the detection of macrometastasis on frozen section prompted immediate conversion to modified‐radical hysterectomy with pelvic lymphadenectomy. This approach allowed for comprehensive surgical staging while enabling timely initiation of adjuvant therapy. In a recently published multicenter, randomized, noninferiority trial (PHENIX), sentinel‐lymph‐node biopsy alone was noninferior to pelvic lymphadenectomy with respect to disease‐free survival in patients with early‐stage cervical cancer and SLN‐negative disease, and was associated with fewer surgical complications [9]. However, the PHENIX‐II cohort, which enrolled SLN‐positive patients, was underpowered to inform clinical practice in this subgroup, leaving the optimal management of patients with positive sentinel nodes—such as the present case—unresolved. The optimal management of patients with negative sentinel nodes and the role of completion lymphadenectomy versus observation require further study. Additionally, the cost‐effectiveness of SNNS compared to routine lymphadenectomy has not been definitively established.

Several limitations of this report should be acknowledged. First, mapping was performed using a single tracer (99mTc‐phytate) only; indocyanine green (ICG) was not used in this case, which limits interpretation of the lymphatic drainage pathway and reproducibility in centers without SPECT–CT capability. Following the regulatory approval of ICG for sentinel lymph node mapping in Japan in February 2026, our institutional protocol has been updated to use either ICG combined with radioisotope or ICG alone for sentinel‐node mapping in cervical cancer. Second, this is a single case report, and the conclusions cannot be generalized.

Future research should focus on identifying which patients are most likely to benefit from SNNS, refining the technique to minimize false‐negative rates by combining the dye method using ICG, and establishing standardized protocols for ultrastaging. Incorporation of immunohistochemical markers and molecular analysis of sentinel nodes may further improve detection of micrometastases.

In conclusion, we report a case of presumed Stage IA2 cervical cancer in which SNNS detected a para‐aortic macrometastasis and an obturator micrometastasis unidentified by preoperative imaging. SNNS with ultrastaging enables accurate metastasis detection, improving postoperative quality of life while facilitating individualized treatment selection.

## Author Contributions

**Yutaka Shiraishi:** writing – review and editing. **Kensuke Sakai:** writing – review and editing. **Tatsuyuki Chiyoda:** writing – original draft, writing – review and editing, visualization. **Hiroshi Nishio:** writing – review and editing. **Wataru Yamagami:** writing – review and editing. **Hatsune Ouchi:** writing – review and editing, writing – original draft, visualization.

## Funding

The authors have nothing to report.

## Disclosure

The authors have nothing to report.

## Ethics Statement

This study was approved by the Institutional Review Board of Keio University School of Medicine, approval number 20070081.

## Consent

Written informed consent was obtained from the patient for publication of this case report.

## Conflicts of Interest

The authors declare no conflicts of interest.

## Data Availability

Data sharing not applicable to this article as no datasets were generated or analysed during the current study.
